# Gastric cancer: predictors of recurrence when lymph-node dissection is inadequate

**DOI:** 10.1186/1477-7819-7-69

**Published:** 2009-09-17

**Authors:** Esther Uña

**Affiliations:** 1 Medical Oncology Service, Clinical University Hospital, Valladolid, Spain

## Abstract

**Background:**

The TNM classification (sixth edition) requires at least 15 lymph nodes to be examined to allow an accurate staging. However, in our environment, only 20% of patients have the recommended minimum of 15 nodes removed.

**Purpose:**

To evaluate clinicopathological predictors of recurrence in patients with gastric cancer undergoing radical resection with an inadequate number of lymph nodes examined.

**Methods:**

101 patients were included in this retrospective cohort. We evaluated age, gender, tumoral location, Borrmann type, Lauren histotype, type of gastrectomy, grade, invasion depth of tumor, lymph node involvement, ratio between metastatic and total number of excised lymph nodes keeping 20% as the cutoff value (LNR) and adjuvant treatment. The association between these variables and recurrence was investigated by using univariate methods and multivariate logistic regression analysis.

**Results:**

Median (range) age was 63 years (44-85). 63% males, 37% females. Median follow-up time for the whole patients population was 36 months (10-104). Median number of lymph nodes retrieved was 6 (0-14). Recurrence: 50 of 101 cases (49,6%); 41 hematogeneus dissemination, 9 locoregional recurrences. The following factors were found to be correlated with the recurrence risk: tumoral location, invasion depth of tumor, lymph node involvement and LNR. A multivariate analysis revealed that depth of invasion [odds ratio (OR) 2.80, 95% confidence interval (CI) 1.03-7.58, P = 0.04] and LNR (OR 2.34, 95% CI 1.05-5.21, P = 0.03) were independent risk factors for recurrences of gastric cancer. Median time to recurrence: 16 months (2-50). 82% of recurrences occurred within the first two years after surgical treatment. The estimated cumulative risk of recurrence at five years: 61% in the whole patients population, with serosal invasion and LNR > and < 20% was 82% and 44%, without serosal invasion 73% and 39% respectively.

**Conclusion:**

Invasion depth of tumor and LNR were independent predictors of recurrence in gastric cancer after potentially curative resection with an inadequate number of lymph nodes examined.

## Correspondence

**Metastasis of the lymph node (MLN) **is one of the most important prognostic factors in gastric cancer [1]. Although Aurello et al. [2] have indicated that the number of nodes necessary to conclude N0 may vary according to the depth of tumor invasion (T), the **TNM **classification (International Union Against Cancer, sixth edition) requires the retrieval and analysis of at least 15 lymph nodes for accurate staging. However, in most cases, the number of nodes dissected is smaller and only 20 to 30% of the patients have the recommended minimum dissection of 15 nodes.

Although previous studies have indicated that the lymph-node ratio (LNR), which is defined as the ratio of the number of MLN to the total number of nodes found during pathological examination, is a powerful independent prognostic factor [3,4] with significant superiority in minimizing "stage migration" for patients with more than 15 nodes evaluated, it remains controversial whether this result is applicable to patients with inadequate staging [5]. Many published studies report different cut offs for LNR, but the consensus value seems to be 20% [6].

We carried out an institutional retrospective study to investigate the relationship between classical clinicopathological features and LNR (using 20% as the cut off value) and the prognosis in patients with gastric cancer who underwent an apparently radical resection but who had an insufficient number of lymph nodes retrieved (and, therefore, were inadequately staged).

One hundred and one patients were included in the study from 2003 to 2006. The median age was 63 years (range, 44-85 years) and 63% of patients were male. The median follow-up time was 36 months (range, 10-104 months) and the median number of lymph nodes analyzed was six (range, 0-14 nodes). Recurrence was detected in 50 out of 101 cases (49,6%), 41 patients presented hematogenous dissemination as the main location (20 of them presented also locoregional recurrence with lymph nodes involved in 13 patients), and nine patients had only locoregional recurrence (see Table 1). The high recurrence rate observed in this population **could be explained by the selection process, which was based on limited lymph node dissection and could lead to an increase in the likelihood of understaging**. Many studies have indicated that the number of metastatic nodes in patients with radically resected gastric cancer increases with the number of lymph nodes removed [7].

**Table 1:** Specific sites of gastric cancer recurrence in our population.

**Locoregional**	**Number of patients**
Lymph nodes	5

Anastomosis	2

Gastric bed	2

**Distant**	**Number of patients**

Peritoneal carcinomatosis	18

Liver	11

Lung	8

Bones	3

Pleura	1

The following factors were correlated with the recurrence risk: tumoral location, invasion depth of the tumor, lymph-node involvement (pN), and LNR.

A multivariate analysis of our data revealed that depth of invasion [odds ratio (OR), 2.80; 95% confidence interval (CI), 1.03-7.58; *P *= 0.04] and LNR (OR, 2.34; 95% CI, 1.05-5.21; *P *= 0.03) were independent risk factors for the recurrence of gastric cancer.

The median time to recurrence was 16 months (range, 2-50 months). Eighty-two percent of recurrences occurred within the first two years after surgical treatment. The estimated cumulative risk of recurrence at five years was 61% in the whole population; in the case of serosal invasion and LNR > and ≤ 20%, this risk was 82% and 44%, respectively; without serosal invasion, the risk was 73% and 39%, respectively with LNR > and ≤ 20%.

Although we did not apply any specific method to select the LNR cut off, we nevertheless obtained an interesting and significant result considering 20% as the cut off most frequently used. It is clear that the fewer the nodes examined, the lower was the accuracy of the prognostic system.

Our study revealed that depth of tumor invasion and LNR with a 20% cutoff value were independent and significant predictors of recurrence in gastric cancer after potentially curative resection with examination of an inadequate number of lymph nodes.

## Competing interests

The author declares that she has no competing interests.
